# Gas phase composition of a NiMH battery during a work cycle†

**DOI:** 10.1039/d4ra02130d

**Published:** 2024-06-21

**Authors:** Aleksandra Lindberg, Björn Eriksson, Jenny Börjesson Axén, Amritha P. Sandra, Göran Lindbergh

**Affiliations:** a Applied Electrochemistry, Department of Chemical Engineering, School of Engineering Sciences in Chemistry, Biotechnology and Health, KTH Royal Institute of Technology SE-100 44 Stockholm Sweden gnli@kth.se; b Nilar AB Bönavägen 55 SE-806 47 Gävle Sweden

## Abstract

Side reactions leading to gas evolution are undesirable in batteries and result in reduced coulombic efficiency and shortened lifetime. Quantitative analysis of the gases that evolve is therefore important to improve understanding of the reactions occurring in the battery during cycling and could be used to optimize battery operation. However, the measurements are challenging because batteries are by their nature closed with limited gas space. Nickel metal hydride (NiMH) batteries are widely used due to their good rate capability, reliability, and environmental friendliness. The battery type has been extensively studied in terms of degradation and performance. However, very few studies have been conducted on the gas composition created during a work cycle. In this study, two methods for investigating the internal NiMH battery gas phase composition during different charge/discharge cycles using a mass spectrometer (MS) were developed. In the first method, the battery module was connected by a sampler system. In the second method, the battery was connected directly using a microcapillary, and the gas composition was continuously measured. In addition to the gas composition, the voltage, pressure, and temperature of the battery were recorded. The most abundant component in the measured gas phase was nitrogen, present in the cell from the assembly stage, followed by hydrogen. A clear rising trend of hydrogen pressure as depth of charge (DOC) increased was recorded, while oxygen levels were low except around the end of charge. The methods were found to be a reliable means of investigating NiMH gas composition without negatively affecting the battery and may be adapted to other battery chemistries.

## Abstract 2

In this study, two methods for investigating the internal NiMH battery gas phase composition during different charge/discharge cycles using a mass spectrometer (MS) were developed.

## Graphical abstract

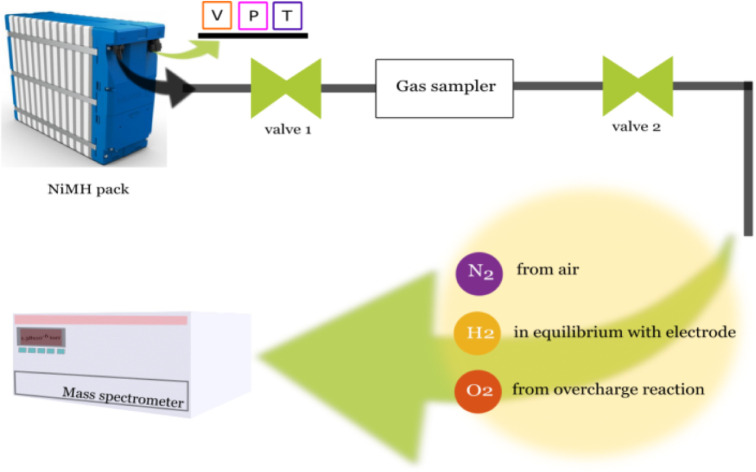

## Introduction

The internal gas composition of batteries is not a common topic of discussion, despite the pivotal role that batteries play in the transition towards a carbon-neutral society, especially considering their application in electrification of the transport sector. The acceptability of battery technologies in any field highly relies on their performance, quality, reliability, sustainability and safety.^1^ Therefore, it is essential to understand the ageing behavior of batteries to gain deeper insights into their performance and safety. The ageing of batteries is a complex phenomenon resulting from parasitic reactions involving solid, liquid, and gaseous reactants and products. Consequently, the identification and quantification of these reactions represents a significant challenge. Among these parasitic reactions, gas generation is one of the detrimental effects in the degradation of batteries, which can induce mechanical stress, increase the internal resistance and reduce the cycle lifetime.^2^ Therefore, it is essential to understand the internal gas composition of batteries. Nevertheless, the analysis of the gas present within batteries is challenging because of the complexities of both chemical and electrochemical reactions that occur during different charge–discharge cycles. Furthermore, the gas consumption has a significant impact on the quantity and constituents of the collected gases.^3^ The nature of gas evolution reactions varies with different battery chemistries *i.e.* the composition of the electrode materials and the electrolytes.^4^ Additionally, the electrode cross-talk has the ability to absorb or consume the gaseous products due to the transport of reaction products between the electrodes. To address these challenges, *in situ* or *operando* techniques such as mass spectrometry are invaluable in addition to electrochemical characterization providing a more comprehensive understanding of these parasitic reactions.^5^

Few studies have focused on investigating the gas composition in nickel metal hydride (NiMH) batteries. The gas phase of the battery contains nitrogen gas, a consequence of the production process of the battery, hydrogen gas, in equilibrium with the intercalated hydrogen in the metal hydride (MH) electrode, and oxygen gas from the overcharge reaction. Additionally, water vapor is anticipated to be present in equilibrium with the aqueous electrolyte. A study by Mank *et al.* employed Raman spectroscopy to monitor the formation of the gases during the charging of a commercial AA-size battery at different charge rates.^6^ The results indicated a pressure buildup within the cell during charging, accompanied by an increase in the partial pressure of all the gases of interest (O_2_, H_2_ and N_2_). These gases contribute to the overall pressure increase within the battery, and that contribution depends on the battery temperature and the current imposed upon it. Another study by Krüsemann *et al.* employed mass spectrometry with the same analytical aim, to follow gas evolution.^7^ With the developed technique, they were able to record the gas composition, revealing that the hydrogen pressure within the battery initially increases during overcharging. However, as the overcharging continues, the oxygen pressure becomes more prominent. Eventually, at the end of the overcharge period, the oxygen pressure surpasses the hydrogen pressure. Despite these promising results, this study was conducted on a commercial AA battery, which is relatively small and easily maintains isothermal conditions. To gain more comprehensive understanding of the processes occurring within a battery utilized for stationary storage applications, it would be beneficial to conduct studies on larger batteries.

NiMH batteries have excellent cycle life and reasonable specific energy, which have made them an attractive and prominent choice for use in hybrid electric vehicles and aerospace applications.^8^ These batteries are also characterized by high storage capacity, good rate capability, reliability, and environmental friendliness.^9^

The NiMH battery consists of a positive Ni(OH)_2_ electrode and a negative metal hydride electrode (MH). The two electrodes are electrically isolated from each other by a separator impregnated with a potassium hydroxide (KOH) electrolyte, which provides the ionic conductivity between the two electrodes. The overall electrochemical reaction can be described as follows, with charge in the forwards direction and discharge in the backwards reaction:1
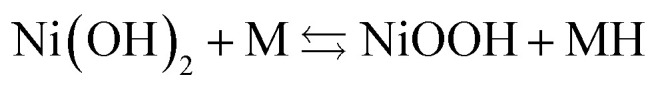


Which can be split into the positive reaction (occurring on the positive electrode):2
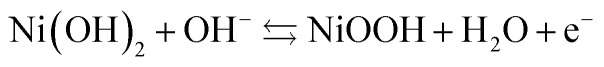
and the negative reaction (occurring on the negative electrode):3
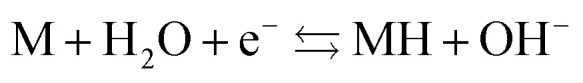


As the battery is charged, hydrogen is transported from the positive to the negative electrode. On the positive electrode, Ni(ii) gets oxidized to Ni(iii) (eqn (2)) while hydrogen ions from the water are reduced to hydrogen atoms at the metal (M) electrode (eqn (3)), which is then absorbed by the hydrogen storage alloy. The hydrogen stored in the MH electrode is in equilibrium with the hydrogen in the gas phase.

To ensure the functioning of the NiMH battery under different conditions, the positive Ni(OH)_2_ electrode has been designed to be the capacity-determining electrode.^10^ This ensures that the high and low charge levels of the metal hydride are avoided, and allows for some corrosion and corresponding loss of capacity of the negative electrode without impacting the cycle life of the battery.

In addition to the main charge and discharge reactions, the battery cell also undergoes a series of side reactions due to the intersection of the cell voltage window with the water splitting reaction potentials. The main product of these side reactions is oxygen gas, which is produced through the oxygen evolution reaction when charging approaches 100% (overcharging):^11^4
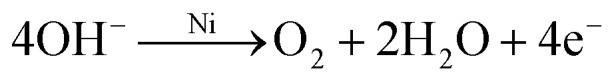


This results in an increase of the partial oxygen pressure inside the cell. Simultaneously, oxygen is transported to the MH electrode, where it is recombined:5
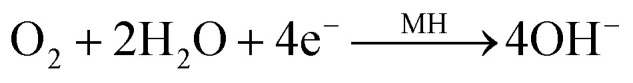


The oxygen recombination mechanism inhibits drastic buildup of internal pressure in the battery cell.^12^ Furthermore, it reforms the water lost from the reaction, thus enabling the battery to remain in a starved electrolyte configuration.

Previous studies have shown that at the end of the charging process a sharp increase in the battery voltage is recorded as the overcharging processes start. This phenomenon is a consequence of the competition on the positive electrode of the oxygen evolution reaction, eqn (4), and the nickel electrode reaction, eqn (2).^13^ This is accompanied by a sharp rise in pressure and temperature as the excess energy is released as heat when the oxygen is recombined.

Although the NiMH battery is commercialized and well-studied, most studies in the available literature have focused on investigating electrode composition. For instance, the review paper by Cuevas summed up the history of development of negative electrode materials for NiMH batteries.^14^ To increase the energy density of the batteries significant efforts were directed towards the development of novel lightweight materials for hydrogen storage.^15^

In our work we analyze the internal gas composition of a 10 A h 10-cell NiMH battery module during different charging cycles by the means of mass-spectrometry. We employ two different setups for this purpose: the utilization of a sample volume together with a standard capillary and the continuous sampling with a microcapillary. The levels of oxygen (O_2_), hydrogen (H_2_) and nitrogen (N_2_) are monitored after charging or discharging a battery to the desired charge/discharge state. Furthermore, the voltage, total pressure and the external battery module temperature are also monitored simultaneously.

## Experimental

### Mass-spectrometry measurements

For all measurements, a NiMH Nilar Energy module was utilized. This battery module consists of ten battery cells, where each cell has positive and negative electrode and separator. The capacity was 10 A h and the nominal voltage 12 V due to the serial configuration of these ten cells, where each cell individually contributes with 1.2 V.

**Fig. 1:**
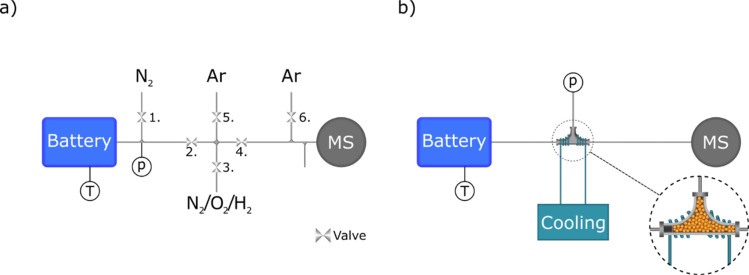
Schematic presentation of the two different experimental setups. (a) The sampler setup, where valves enabled to sample the battery gas phase once per cycle. (b) The microcapillary setup, where gas is continuously sampled using a microcapillary. The gas is cooled and dried before sampled by the capillary to avoid water condensation and clogging of the system.

The cells are arranged horizontally one on top of the other one, in a bipolar configuration with a common gas space.^16^ The module has been modified with an exhaust tube connected to the common gas space. This allowed the quantification of gases, as illustrated in Fig. 1a. To monitor the internal gas pressure, a pressure sensor (SSI Technologies Inc.) was placed in a three-way junction connected to the exhaust tube. While the internal cell temperature could not be monitored, the outer casing of the top cell was monitored using a T-type thermocouple. The supply of gases was regulated by the use of two mass flow controllers (Alicat and Bronkhorst).

In the first setup, Fig. 1a, the exhaust tube from the battery was connected to a system with 6 valves (ASCO) to allow gas sampling, while avoiding depleting the battery of gas. The system allows for batch sampling of a given volume between valves 2,3,4 and 5. The pipe system made of Swagelok elements was thermally isolated and heated up to 50 °C in order to avoid water condensation. During the test cycles valve 2 was open, allowing gas from the battery to flow through the pipes to the sample chamber. Valve 6 was also open, allowing a constant flow of Ar to the mass spectrometer (MS), in order to keep a constant gas intake for the MS. Once a sample has been taken valves 2 and 6 were closed, and valves 4 and 5 were opened, allowing the gas in the sampler to be carried by an Ar flow of 20 ml min^−1^ to reach the MS. For all measurements, a residual gas analyzer (RGA) from Hiden (model HPR-20) was utilized. The sampling time was one hour to ensure that all of the gas from the battery was measured. Between each sampling, the sampler was first flushed with N_2_ by closing valve 4 and opening valve 3 to a flow of N_2_. This step is important to not contaminate the battery with any remaining Ar in the sampler during the next cycle. Prior to starting another cycle, valve 1 was opened to refill the battery with N_2_ to a pressure of 1.1 bar, thus ensuring that gas depletion did not occur as a consequence of the sampling procedure.

The MS calibration was conducted using the same setup, where valves 1, 2 and 6 were closed and valve 4 was opened to supply gas (N_2_, O_2_ or H_2_) to the MS. The flows of the calibration gases (N_2_, O_2_ or H_2_) and Ar were varied, see Table S1.† A typical calibration curve of the MS is presented in Fig. S1.† Each combination of the calibration gases was monitored for 20 min, with a stable response. As the mixture of oxygen and hydrogen gas can, under certain instrumental settings like high Source Cage voltage of the MS, record higher water signal at the expense of the oxygen signal, a calibration with this gas mixture was performed (Fig. S2†). The recorded values indicated a higher water amount and a lower oxygen signal than anticipated. This suggests that the actual oxygen content was higher than that recorded by the MS. The results were subsequently corrected based on the aforementioned calibration, with the details of this correction provided in Fig. S3.† The calibration showed that the hydrogen signal is not significantly influenced by the presence of oxygen. Nevertheless, with low Source Cage voltage of the MS, this feature was not recorded, as illustrated in Fig. S4.†

The second part of the measurement was the direct quantification of gases from the cell, shown in Fig. 1b. In this setup, the battery module exhaust tube was connected directly to the mass spectrometry with a microcapillary inlet, which permitted the gases to enter the mass spectrometer without dilution by carrier gas. In order to prevent moisture interference with the gas evolution data and the probable occurrence of capillary blockage, a T-junction filled with silica gel and cooled using 20 °C water was introduced between the microcapillary inlet and the battery pack. This allowed for the condensation and adsorption on the silica gel of nearly all the moisture content flowing from the cell during testing. Similar to the first setup, a thermocouple and a pressure sensor were used to measure the temperature and pressure during operation. The pressure sensor was connected to the T-junction, and the thermocouple was inserted into the battery casing in the same way as in the first setup. This setup allowed for continuous measurement of gases during cycling, with a microcapillary inlet gas intake of 12.5 μl min^−1^. The implementation of this particular configuration served to improve the precision of our gas analysis methodology.

The calibration of the measurements of the second setup was conducted by connecting the flow controllers to the T-junction, replacing the battery module. Two flow controllers were employed to calibrate two gas mixtures: oxygen with nitrogen and hydrogen with nitrogen. Two factors had to be calibrated for: the gas composition, and the total gas pressure. As the gas is transferred directly from the cell to the mass spectrometer, any fluctuations in pressure within the cell also affect the pressure at the inlet of the mass spectrometer. The calibration of O_2_/N_2_ mixture was done by altering the oxygen gas percentage (0%, 8.4%, 21.1%, 30.3%, 50%) in the mixture with the help of flow meters and maintaining the total flow to 200 ml min^−1^ (Fig. S5a†). In addition, the pressure was also monitored using the flow controllers and an external pressure sensor. The second calibration was conducted by varying the total pressure (1.0 bar, 1.5 bar, 2.0 bar, 2.5 bar) of the O_2_/N_2_ mixture with the same compositions as measured in the first calibration step (Fig. S5b†). In the all the O_2_/N_2_ mixtures, the signals were proportional to the gas composition, independent of the total pressure. The calibration of hydrogen was done with different gas compositions and total pressure, as shown in (Fig. S6a†). However, the H_2_/N_2_ mixture showed a different behavior to the O_2_/N_2_ mixture. The total pressure calibration curve, (Fig. S6b†), does not follow a linear behavior and the signal ratio changes for different compositions. Therefore, a non-linear function was utilized for fitting. The data obtained using the microcapillary were corrected using the resulting calibration functions. This behavior may be a result of increasing pressure reducing the mean free path for larger molecules, that can affect the signal. Furthermore, the pumping efficiency and the ionization efficiency of the MS are pressure dependent.

### Electrochemical measurements

To test the battery internal gas composition using the first setup, 11 different sampling cycles were chosen, given in Table 1. For all measurements the battery was charged with 0.25C and discharged with 0.2C, where C-rate is defined as the inverse of the theoretical time needed to fully discharge the battery by employing constant current. In cycles 1–7 the cell was charged to the given depth of charge (DOC) prior to gas sampling. DOC represents the ratio between how much capacity has been charged into the battery and the total capacity of the battery, independent on side reactions. For cycles 8–11 the battery was first charged to 110% DOC (similar to cycle 7) and then allowed to rest at open circuit potential prior to sampling. For cycle 10 and 11 the battery was first charged to 110% DOC, allowed to rest for 1 h and then discharged to the indicated depth of discharge (DOD). DOD represents the ratio between how much capacity has been discharged and the total capacity of the battery. After sampling, the battery was allowed to rest for 1 h before being discharged to a cell voltage of 1 V, and then allowed to rest for 2 h before starting another cycle. The electrochemical measurements were done using a PGSTAT302N (Metrohm) with a 10 A booster, while the cell voltage was continuously monitored using a multimeter (Keithley).

**Table 1:** Test cycles used for the sampler measurements. Charge % in DOC, discharge % in DOD

Cycle	Operations	Name
1	Charge	10.4% DOC
2	Charge	50% DOC
3	Charge	80% DOC
4	Charge	100% DOC
5	Charge	104% DOC
6	Charge	108% DOC
7	Charge	110% DOC
8	Charge + rest	Step 7 + rest 5 min
9	Charge + rest	Step 7 + rest 55 min
10	Charge + rest + discharge	Step 9 + discharge 3% DOD
11	Charge + rest + discharge	Step 9 + discharge 90% DOD

## Results and discussion

**Fig. 2:**
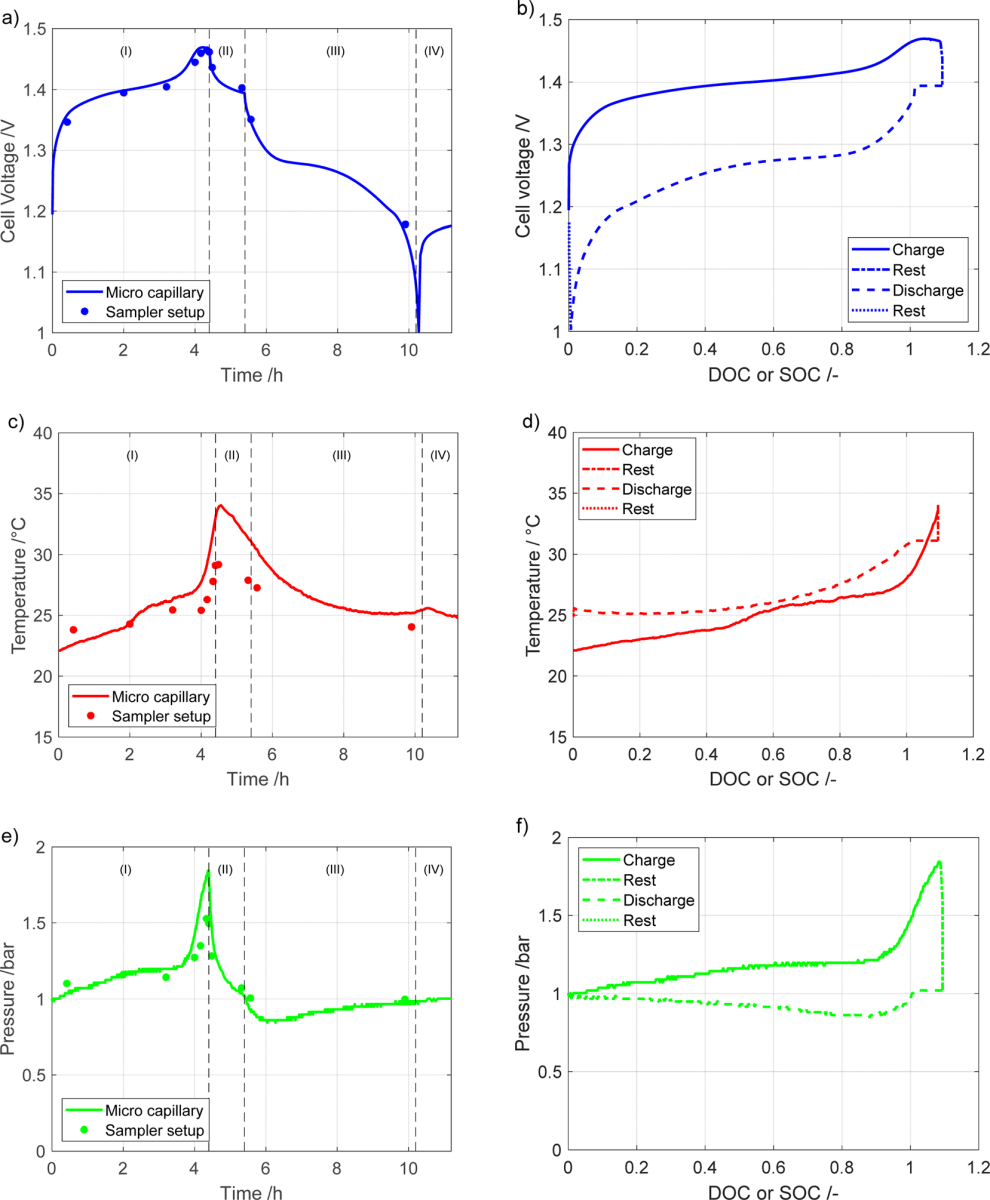
Cell voltage (a and b), temperature (c and d), total pressure (e and f) signals over time (a, c and e) and as a function of DOC (charge) or SOC (discharge) (b, d and f). Measurements done at room temperature and with a charging rate of 0.25C and discharge rate of 0.2C.

In Fig. 2 the voltage, temperature, and pressure behavior of the module during the cycles are presented. The data are obtained by two experimental configurations: the sampler setup, represented by dots, and the microcapillary set-up, represented by the solid line. The steps listed in Table 1 can be observed in the measurements, with four clearly discernable regions: (I) charging, (II) rest, (III) discharge and (IV) rest.

As can be seen during the constant current charging region (I) the temperature, voltage, and pressure rise for both types of measurements. Above 100% DOC, the temperature, voltage and pressure all increase more rapidly until the charging is stopped after 4.2 h. A time delay in the temperature response, Fig. 2a, compared to the voltage and pressure can be observed. This delay is most likely due to the thermocouple being placed on the upper casing of the module, meaning there is a delay between the generation of heat in the cells and the measurement on the casing.

During the first rest step, (II), the voltage decreases rapidly, but eventually stabilizes. This is due to typical self-discharge behavior of NiMH batteries as well as battery relaxation, where gradients built up during charging are relaxed. As it is indicated by the fast nature of the voltage decrease, it is mostly due to continued oxygen evolution caused by the high voltage on the positive electrode at the end of charge. Simultaneously, with the voltage reduction, there is a significant reduction in pressure. This is partly due to hydrogen partial pressure decreasing from self-discharge, both from the decrease in hydrogen content and the resulting decrease in temperature, and partly due to the oxygen recombination mechanism on the negative electrode. During the constant current discharge (III) the cell voltage decreases gradually, before rapidly dropping to 1 V, which signifies a complete discharge. Initially during discharge the pressure dropped quickly, before stabilizing at a value of approximately 0.96 bar. The temperature decreases similarly during the first rest (II) and discharge (III) steps. During discharge, the endothermic deintercalation reaction on the negative electrode causes the battery temperature to decrease. The slight temperature difference between the start and the second rest (IV) is most likely due to the environmental temperature not being controlled, and not an effect of cycling. The measurements shown in Fig. 2 are in line with the previous measurements of a NiMH battery.^9,10^ While the results from the two different experimental setups are similar electrochemically, there are some differences. The most significant difference in the results for the two setups is in the temperature, as a higher temperature is recorded for the microcapillary setup than for the sample setup. A difference in the total pressure is also noted, with the microcapillary setup reaching higher gas pressures, most likely due to the smaller additional gas volume when using this setup.

### Gas composition measurements

**Fig. 3:**
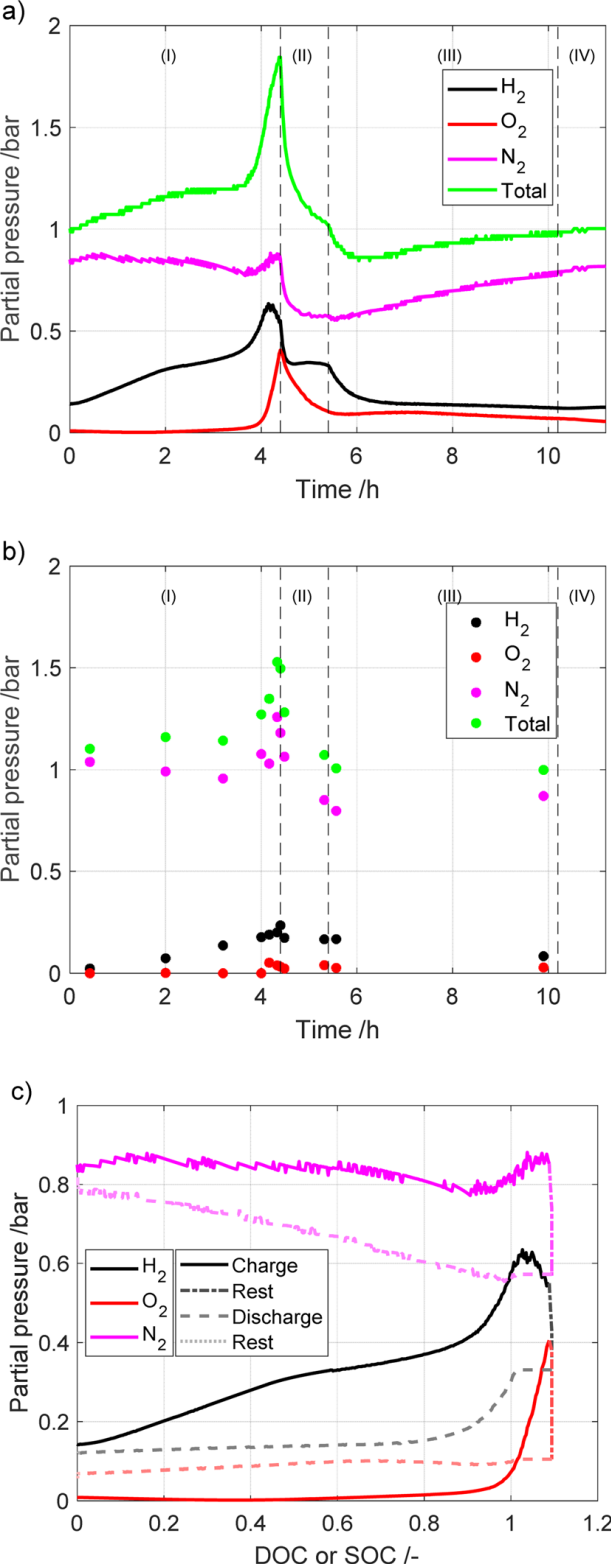
Partial pressures measured using the (a) microcapillary setup, *vs.* time; (b) sampler setup *vs.* time; (c) microcapillary setup, *vs.* DOC/SOC.

The results from the gas-composition measurements from the two experimental setups are presented in Fig. 3. As mentioned earlier, for the sampler setup 20 ml per min Ar was used as a carrier gas to be able to quantify the measured gases. As such, all of the presented data are normalized using the Ar signal, and the Ar signal is not shown.

The highest partial pressure of the gases from the battery pack is that of N_2_, which is dominant, followed by that of H_2_ and O_2_, in that order. The measured N_2_ is from the air that is present in the battery during assembly. The large quantity of H_2_ is most likely due to the equilibrium between the metal hydride and the gas, where the partial pressure is proportional to both degree of intercalation and temperature. The amount of H_2_ is affected by corrosion of the negative electrode material, with a more corroded (*i.e.* aged) electrode releasing more H_2_. As expected, the amount of H_2_ increases with increasing DOC, as the temperature and intercalation degree increase. The detection of an oxygen partial pressure only occurs after charging for more than 4 hours (around 100% of nominal DOC). This is expected, since the oxygen evolution reaction takes place at elevated voltages on the positive electrode. Surprisingly, the measured O_2_ partial pressure was significant even after the battery was discharged and left to rest. This is most likely due to the oxygen recombination being slow. In the sampler setup, the last detected signal is the water pressure, which is low and mostly constant over the cycle. No water signal was detected for the microcapillary set-up, as the water was removed to avoid clogging of the capillary.

**Fig. 4:**
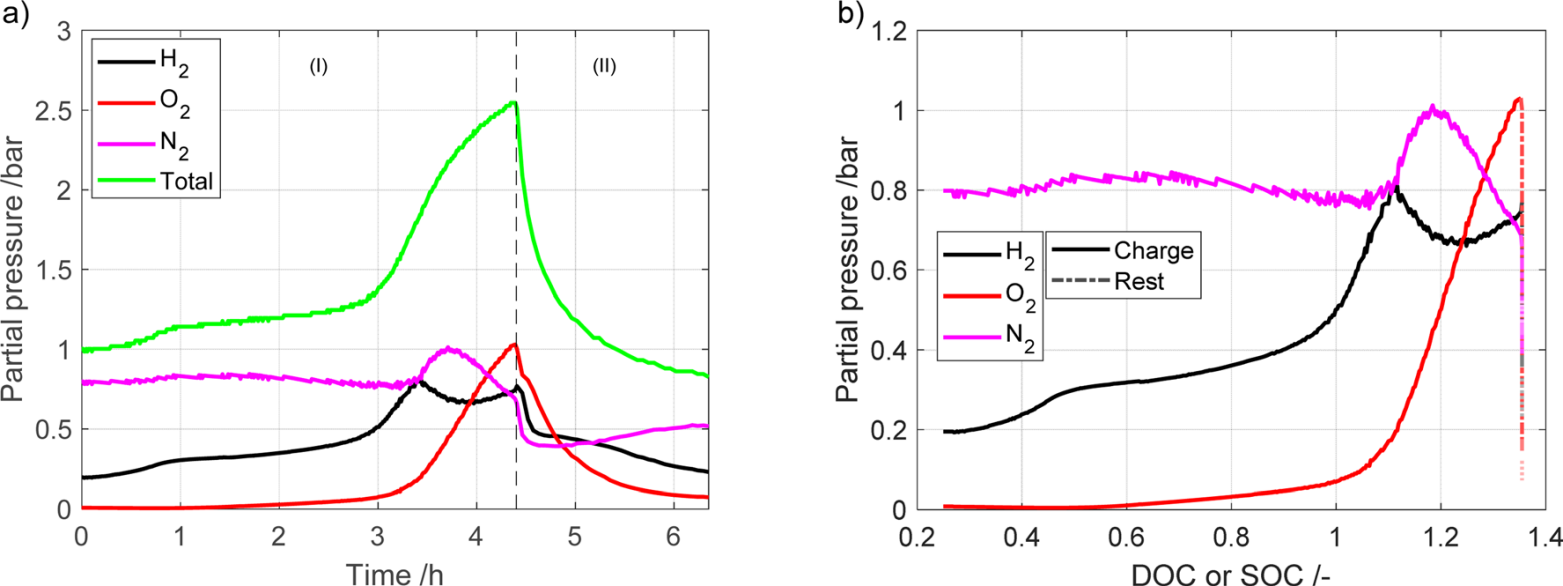
Partial pressures during charging and later overcharging the battery module *vs.* (a) time (b) DOC or SOC.

To further study the gas evolution a measurement with a long period of overcharge was performed using the microcapillary setup. The measured partial pressures are shown in Fig. 4. The cell voltage, pressure and temperature are given in Fig. S7.† In Fig. 4a, the region marked as (I) represents the battery charging process, while region (II) presents the rest period. As the battery enters into overcharging territory (after *ca.* 3 h), the oxygen evolution side reaction takes over from the charge reaction, leading to oxygen becoming the largest contributor of the detected gases. Similar to the other measurement, the total pressure drops significantly after charging has stopped.

This study showed that diverse experimental setups generate different results that complement each other while illustrating similar patterns. Utilizing a microcapillary configuration led to the observation that the change in pressure altered the ratio of hydrogen to nitrogen gas in the mass spectroscopy measurements. This, in turn, led to the development of an empirical calibration curve, as detailed in the ESI.† Conversely, the sampler setup detected a higher, so-called “phantom” signal of water due to the simultaneous recording of hydrogen and oxygen. The setups also differ in the sampling volume. It is worth noting that ambient temperature was not controlled during the experiments, and the measurements were conducted in different seasons. This variability in temperature could be a contributing factor to the differing hydrogen concentrations between the setups, as hydrogen equilibrium pressure is temperature-dependent. Additionally, the battery module age may also have influenced hydrogen generation. However, the recorded voltage is very similar between the two setups, suggesting that the aging of the module between tests is insignificant.

All three major gases (nitrogen, oxygen and hydrogen) are involved in the pressure buildup in the battery. However, their individual contribution is dependent on temperature and on the currents to which the battery is exposed. In a previous study by Mank *et al.*^6^ that used Raman spectroscopy to analyze gas composition of the NiMH battery, it was found that once the battery was overcharged, the internal temperature increased. This in turn led to the oxygen pressure increasing up to 8 bar. Interestingly, the hydrogen pressure also increased, but to a much lower level, only 2 bar. However, in the experiments where the temperature was held constant, hydrogen was the major contributor to the gas phase. As they used a different electrode material in the battery, we cannot compare the absolute values of the results of this study with our study, as different metal hydride materials have different equilibrium pressure profiles.^17^ However, looking at the trends it is clear that in our study the opposite trends are found with a higher partial pressure of H_2_ compared to O_2_ until 125% DOC. This could be due to different factors, such as the individual metal hydride used in the respective batteries having different equilibrium pressure profiles.

The findings in our research align more closely with the study conducted by Krüsemann *et al.*^7^ They record that, during overcharging, the hydrogen concentration dominates over oxygen, with the positions switched at the end of overcharging. Upon comparing the outcomes obtained through mass spectrometry and Raman spectrometry, their conclusion is that a higher rate of hydrogen release occurs at elevated C-rates.

Overall, the consistency between different measurements in this study and the explainable behavior of the gases prove that the methodology developed in this study is capable of capturing and measuring the gas phase of a NiMH battery with reasonable results.

## Conclusions

In this study, the gas composition of a NiMH battery during a charge and discharge cycle was measured using mass spectroscopy. Two methods have been developed for this purpose: the sampler method and the capillary method. The results of the study show that both methods yielded comparable qualitative results. The analysis revealed that nitrogen was the predominant component of the gas phase, exhibiting a relatively stable pressure profile, with the exception of a minor peak observed at the end of the charge period, followed by a pressure trough with its lowest point observed at the beginning of the discharge period. Hydrogen was the second most prevalent gas with a slow pressure increase during charge that turns more rapid towards the end of charge, due to increased temperature and degree of intercalation. During discharge the pattern is reversed. Oxygen was detected first at the end of charge and during overcharge, which is due to the oxygen evolution reaction at the positive electrode. During the rest step and the following discharge, the oxygen pressure decreased but not entirely.

While the findings were qualitatively synchronized, there were quantitative distinctions in the results obtained from these two methods, which could be accounted for by differences in their setups. The results also agree with previous studies and theory. The choice of method therefore depends on the battery chosen and application studied. A battery with a small free gas volume would be negatively affected by the constant gas consumption by the capillary method, and more accurate results would therefore be produced by the sample method. On the other hand, a test with a complex charge/discharge pattern would need many samples to map out, therefore favoring use of the capillary method.

By using a mass spectrometer to measure the internal gas composition of the NiMH battery, a deeper understanding of the internal gas mechanisms of the NiMH battery can be reached. This knowledge is valuable as the pressure and composition of the gas phase are important factors of the function and aging of NiMH batteries. The methodology that has been developed in this work can also be useful for studies of other battery systems.

## Conflicts of interest

There are no conflicts to declare.

## Supplementary Material

RA-014-D4RA02130D-s001
