# Supplementary material for: Gas phase composition of a NiMH battery during a work cycle
Source: RSC Adv. 2024 Jun 21;14(28):19996–20003. doi: 10.1039/d4ra02130d (PMC11191050; doi:10.1039/d4ra02130d)
Supplement: RA-014-D4RA02130D-s001 [file RA-014-D4RA02130D-s001.pdf]

Electronic Supplementary Material (ESI) for RSC Advances.
This journal is © The Royal Society of Chemistry 2024

Supporting information for

Gas Phase Composition of a NiMH during a work cycle

Aleksandra Lindberg[1], Björn Eriksson[1], Jenny Börjesson Axen[1,2], Amritha P Sandra[1], Göran Lindbergh[1]*

[1] Applied Electrochemistry, Department of Chemical Engineering, School of Engineering Sciences in Chemistry, Biotechnology and Health, KTH Royal Institute of Technology, Sweden

[2] Nilar AB - – Bönavägen 55, 806 47 Gävle, Sweden.

* gnli@kth.se

MS calibration sampler

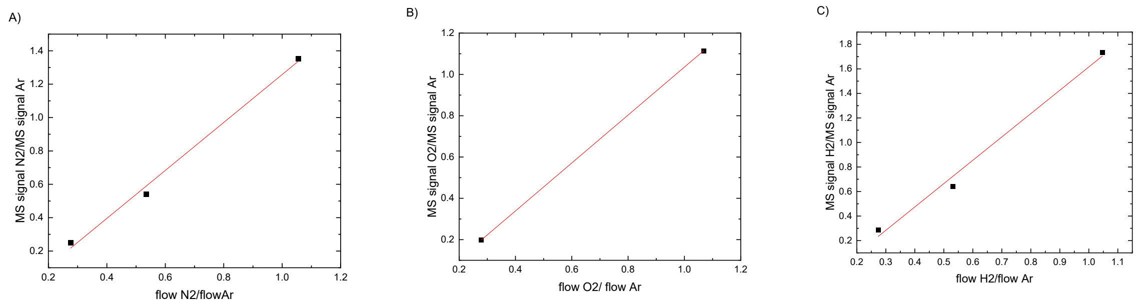

Figure S1. The calibration curves for A) nitrogen, B) oxygen and C) hydrogen, normalized by argon signal.

Table S1. Flow rates of the calibration gasses individually mixed with Ar

	N_2	H_2	O_2	Ar
Gas/	20.6	20.5	20.9	19.1
Flow	11.1	11.0	11.3	19.1
(ml min^{-1})	6.4	6.3	6.5	19.1

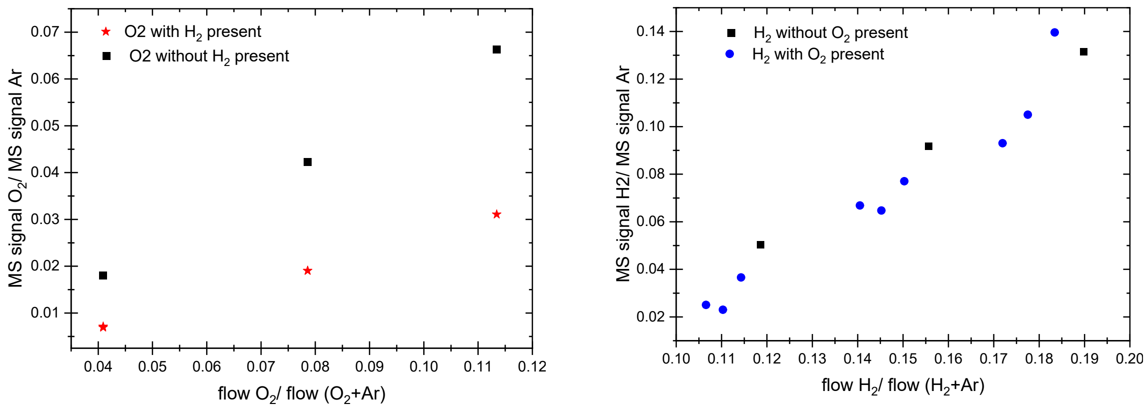

Figure S2. MS calibration curve obtained with O_2 and H_2 mixture, with 20 ml min^{-1} Ar flow, as a

carrier gas.

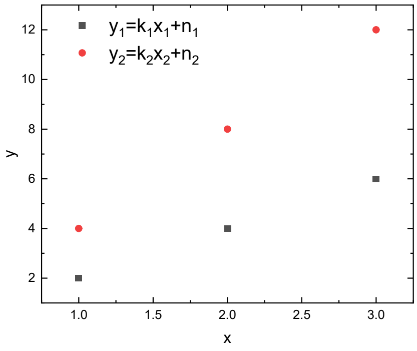

Figure S3. General example of the situation given in the Figure S2.

Figure S3 presents general case showing two data sets, similar to the Figure S2 A). To correct for this deviation y_2 can be calculated as:

$$y_2 = k_2\left(\frac{y_1 - n_1}{k_1}\right) + n_2$$

Real oxygen signal is in that case:

$$O_2 = \frac{\frac{O_2}{Ar} + n_2}{k_1 * k_2 - n_1}$$

And water:

$$H_2O = \frac{H_2O}{Ar} - (k * \frac{O_2}{Ar} + n)$$

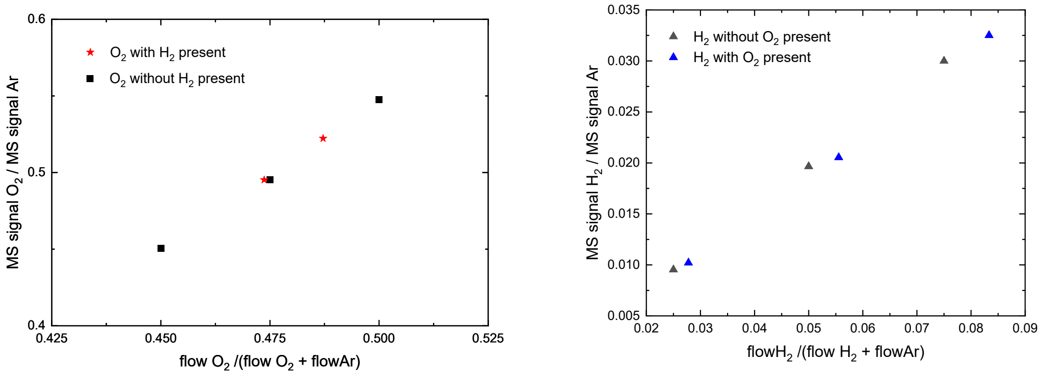

Figure S4. MS response when measuring O_2 and H_2 mixture, with Ar as a carrier gas

MS calibration microcapillary

For either H_2 or O_2 with N_2 calibration was performed by using two mass flow controllers and sampling the stream. A backpressure regulator was used to investigate if the pressure affected the signal. For all measurements a total flow of 200 ml/min was used.

O_2 with N_2 calibration

As can be seen in Figure S5 the MS signal follows the set dilution well. When the total pressure is increased the total signals for both O_2 and N_2 were increased. However, the proportion of the two signals remained constant.

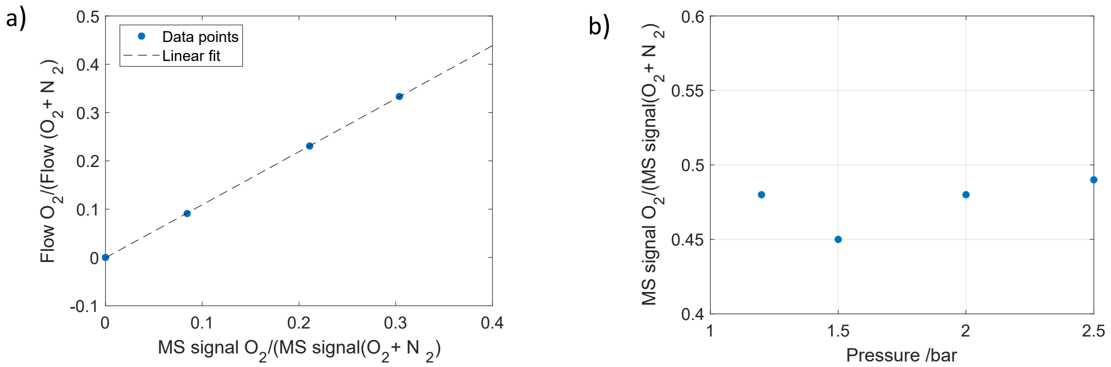

Figure S5. a) MS calibration curve obtained with O_2 and N_2 mixture at atm. (b) Raw MS signals compared at various total pressures. The set flow was 100 ml/min O_2 and 100 ml/min N_2.

H_2 with N_2 calibration

For H_2 with N_2 linear increases with dilution where obtained. However, as the pressure was increased the obtained ratio changed. As such, a different type of calibration was required. Considering how the signal for the same dilution changed with pressure the following function was utilized:

$$f(p_{abs}) = \frac{k_1}{\left(1 + e^{-k_2 * p_{abs}}\right)^{k_3}} + k_4$$

Where k are constants and p_{abs} is the absolute pressure of the system.

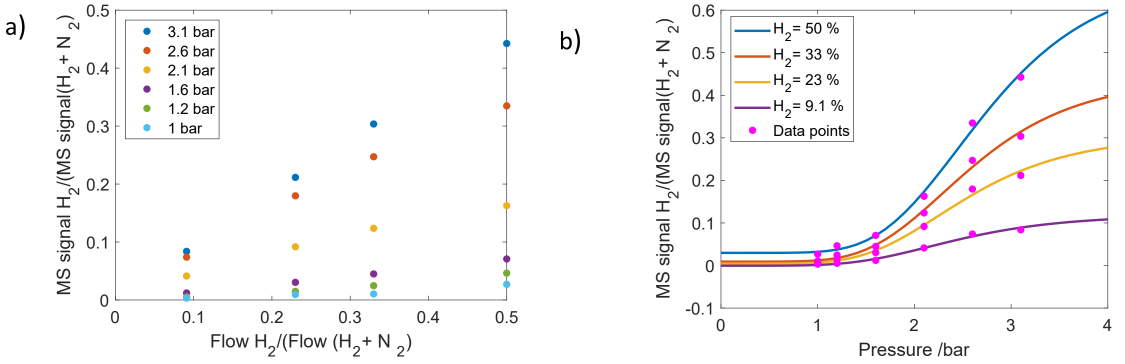

Figure S6. MS calibration curve obtained with H_2 and N_2 mixtures. A) MS signals compared to the set composition. B) MS signals for different gas compositions at various total pressures.

Using these imperical functions a calibration curve is made for each measured point and converted to the percentage.

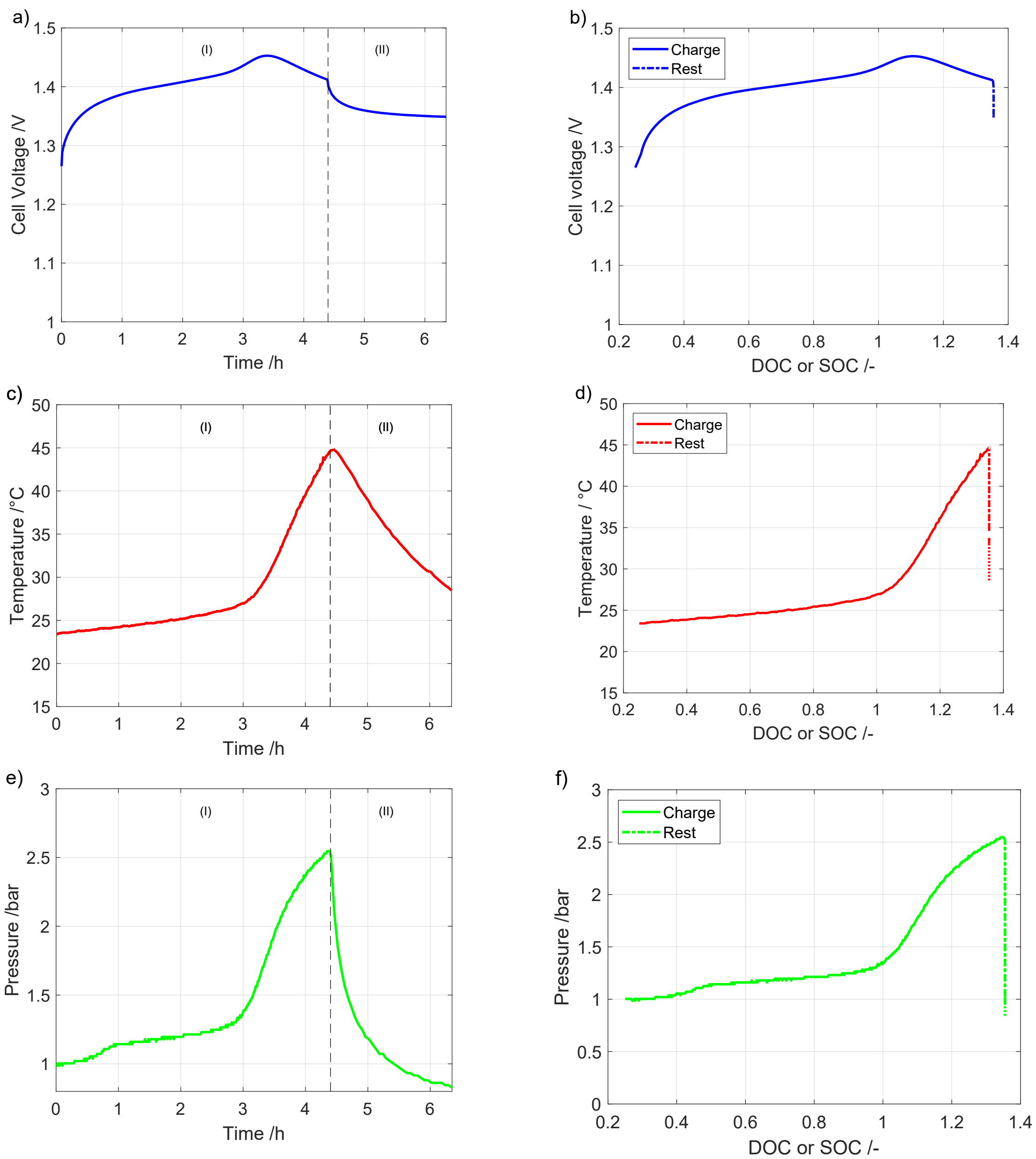

Figure S7. Cell voltage (a,b)/ temperature (c,d) and pressure (e,f) during overcharging the battery modul vs time (a,c,e) and DOC or SOC (b,d,f)
